# Q&A: How does peptide signaling direct plant development?

**DOI:** 10.1186/s12915-016-0280-3

**Published:** 2016-07-08

**Authors:** Maike Breiden, Rüdiger Simon

**Affiliations:** Institute for Developmental Genetics, Heinrich-Heine-Universität Düsseldorf, University Street, D-40225 Düsseldorf, Germany; Cluster of Excellence on Plant Sciences and Institute for Developmental Genetics, Heinrich-Heine University, University Street 1, D-40225 Düsseldorf, Germany

## Abstract

A significant part of the communication between plant cells is mediated by signaling peptides and their corresponding plasma membrane-localized receptor-like kinases. This communication mechanism serves as a key regulatory unit for coordination of plant growth and development. In the past years more peptide–receptor signaling pathways have been shown to regulate developmental processes, such as shoot and root meristem maintenance, seed formation, and floral abscission. More detailed understanding of the processes behind this regulation might also be helpful to increase the yield of crop plants.

## How were plant peptides discovered?

Growth and development of multicellular organisms is coordinated via cell-to-cell interactions. Different hormones, including small secreted polypeptides, maintain this communication in plants, fulfilling a vast variety of functions in plant growth, development, and stress responses. Their involvement in developmental processes by acting as a key component in cell-to-cell communication will be the focus of this Q&A.

The first described plant signaling peptide was tomato systemin (TomSys), which was discovered in the 1970s by Clarence E. Ryan [1]. Wounded tomato leaves, when added in water to young tomato plants, induced the production of proteinase inhibitors I and II, and this led to the identification of an 18 amino acid signaling peptide termed TomSys. TomSys is involved in the production of jasmonic acid, which is the main wound response signal, initiating, for example, intracellular signaling cascades via mitogen activated protein kinases (MAPKs) [2, 3].

## What signaling peptides are now known?

Approximately 13 plant peptide families have been identified so far, including CLE (CLAVATA3/EMBRYO-SURROUNDING REGION, CLV3/ESR) and IDA (INFLORESCENCE DEFICIENT IN ABSCISSION), with more than 1000 genes encoding putative small signaling molecules [4]. They can be grouped into two classes, the small post-translationally modified peptides and the cysteine-rich polypeptides [5]. Small post-translationally modified peptides are usually composed of 5 to 20 amino acids. Cysteine-rich polypeptides have a length of approximately 50 amino acids and are synthesized as precursor proteins [6]. Peptide families that belong to the small post-translationally modified peptides are, for example, the CLE, IDA, and RGF (ROOT GROWTH FACTOR) peptides [7–10]. RALF (RAPID ALKALINIZATION FACTOR) and PDF (PLANT DEFENSIN) peptides are examples of cysteine-rich polypeptides [11, 12].

## How are plant peptides processed and post-translationally modified?

**Fig. 1:**
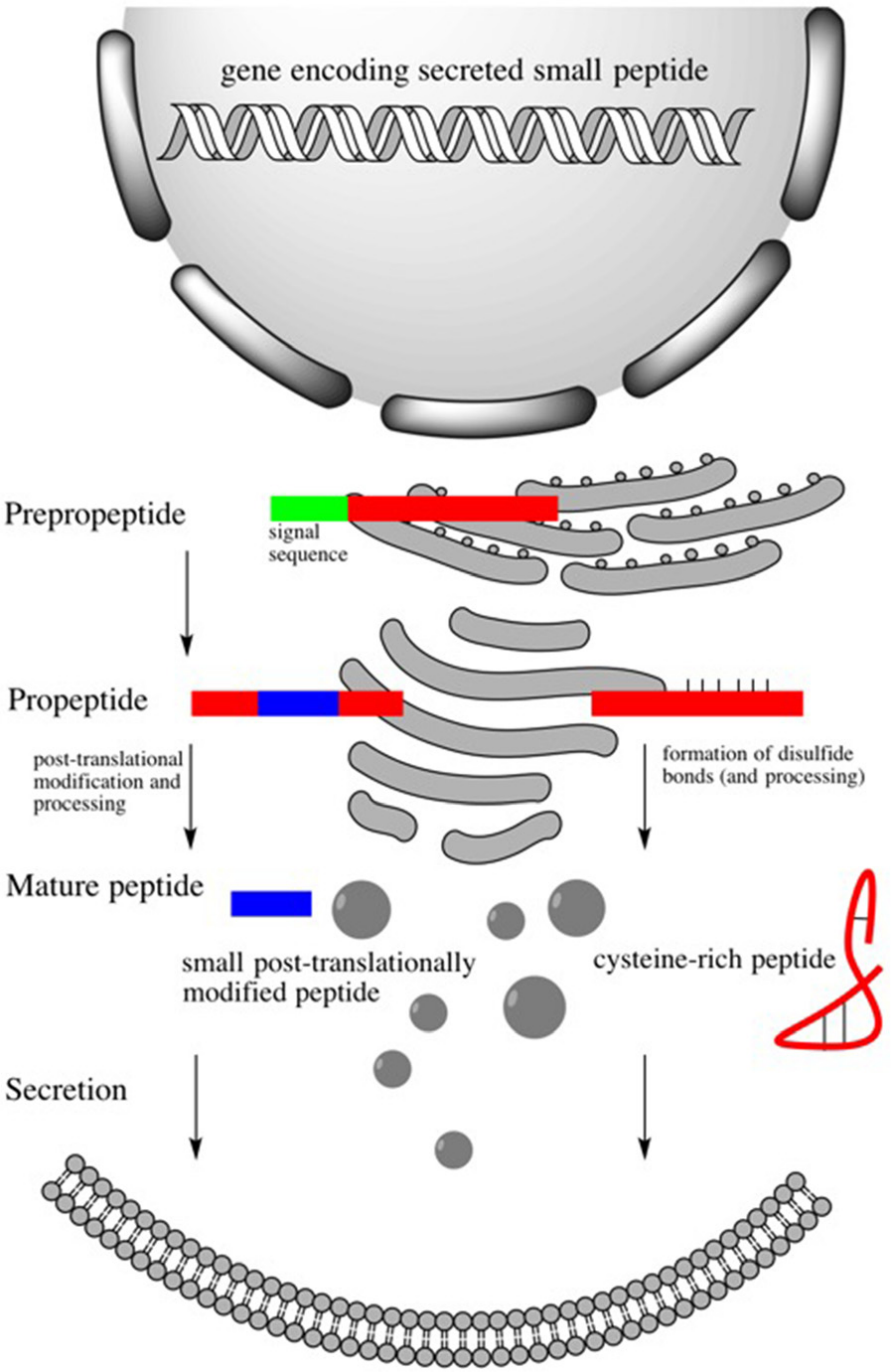
Signaling peptides are mainly synthesized as prepropeptides. First their signal sequence for secretion is cleaved upon entry into the endoplasmic reticulum. Second, post-translational modifications of small peptides and the formation of disulfide bonds of cysteine-rich peptide take place within the Golgi network. The final processing to form the mature peptide can occur in the vesicular transport system or finally in the apoplast and involves proteolytic cleavage, hydroxylation, and arabinosylation

Most plant peptides are products of proteolysed precursor proteins [13]. A few peptides are produced by non-ribosomal synthesis, such as glutathione and phytochelatins [14]. Processing of the precursor peptide can occur in the cytosol or in the apoplast since proteases are also part of the plant secretome [15]. An overview of the processing and secretion of some signaling peptide families is shown in Fig. 1.

For example, members of the CLE peptide family are translated as a prepropeptide with a length of approximately 100 amino acids and are further processed to a 12–14 amino acid peptide [16–18]. The prepropeptides share an N-terminal signal sequence to direct them into the secretory pathway and the conserved CLE motif close to their C-terminus [19–21]. Processing is achieved by serine proteases, most likely members of the subtilisin family that cleave off the N-terminal part of the proprotein at a conserved arginine in the CLE motif. The C-terminal part is then removed by a carboxypeptidase [22, 23]. CLE propeptides are further post-translationally modified by hydroxylation and glycosylation. These modifications are mediated by enzymes during their secretion and enhance the receptor binding activity of the mature CLE peptides [24].

Cysteine-rich peptides usually have an N-terminal signal sequence to direct them into the secretory pathway. Only some of them are further proteolytically processed, such as STOMAGEN, the EPF peptides, and RALF. However, correct folding and establishment of disulfide bridges is required for their function. RALF was first identified in tobacco and RALF23 was shown to be processed by a subtilisin protease [25, 26]. STOMAGEN and the related EPF-like peptides are involved in epidermal patterning and stomatal development and are also processed from larger precursors [27].

Small post-translationally modified peptides carry tyrosine sulfations, proline hydroxylations, or arabinosylations [28]. Some of these modifications change the peptide conformation—for example, the hydroxylation of a proline side chain induces a kink into the peptide that could enhance its affinity for the receptor [29]. The peptides might also be protected from proteolysis by masking the recognition sequences of proteases in their sequence [30].

Tyrosine sulfation is mediated by the tyrosylprotein sulfotransferase (TPST). TPST catalyzes the transfer reaction of sulfate from 3’-phosphoadenosine 5’-phosphosulfate (PAPS) to a tyrosine side chain [31]. Three peptide classes with tyrosine sulfation are known: PSKs (phytosulfokines), PSY1, and RGFs (root meristem growth factors) [9, 32, 33].

Proline hydroxylation is mediated by prolyl-4-hydroxylase (P4H), which is a membrane protein that localizes to the Golgi and endoplasmic reticulum network [34]. Hydroxyproline (Hyp) residues are present in several small post-translationally modified peptides like CLV3, CLE2, and TDIF (tracheary elements differentiation inhibitory factor) [18, 35].

In several small secreted peptides Hyp residues are further linked to an O-linked-I-arabinose chain. Examples of arabinosylated peptides are CLV3 and CLE2 [18].

## How are plant peptides perceived by the cells?

Signaling peptides are perceived via plasma membrane-localized receptor-like kinases (RLKs) [36]. The largest subfamily of RLKs, leucine-rich repeat RLKs (LRR-RLK), consist of more than 200 members in *Arabidopsis* [24]. LRR-RLKs carry an extracellular LRR domain, a transmembrane domain, and an intracellular kinase domain, which is activated upon peptide binding to the LRR domain [37]. The activated kinase domain can induce several different pathways leading to cell growth, proliferation, differentiation, or a defense response.

Five different crystal structures of peptides (PSK, IDA, FLG22, PEP1, TDIF) bound to their receptors (PSKR, HAE, FLS2, PEPR1, PXY, BAK1, SERK1) have been resolved [29, 38–41]. All show the binding of the peptide to inner surfaces of the LRR domains. In all structures the peptides share a similar orientation, with their C-termini pointing towards the C-termini of their respective receptors. The C-termini of FLG22 and PEP1 receptors mediate receptor interactions. Since their binding mechanism is quite similar, this might also be true for the TDIF receptor, PXY [29].

## So what do plant peptides do?

**Fig. 2:**
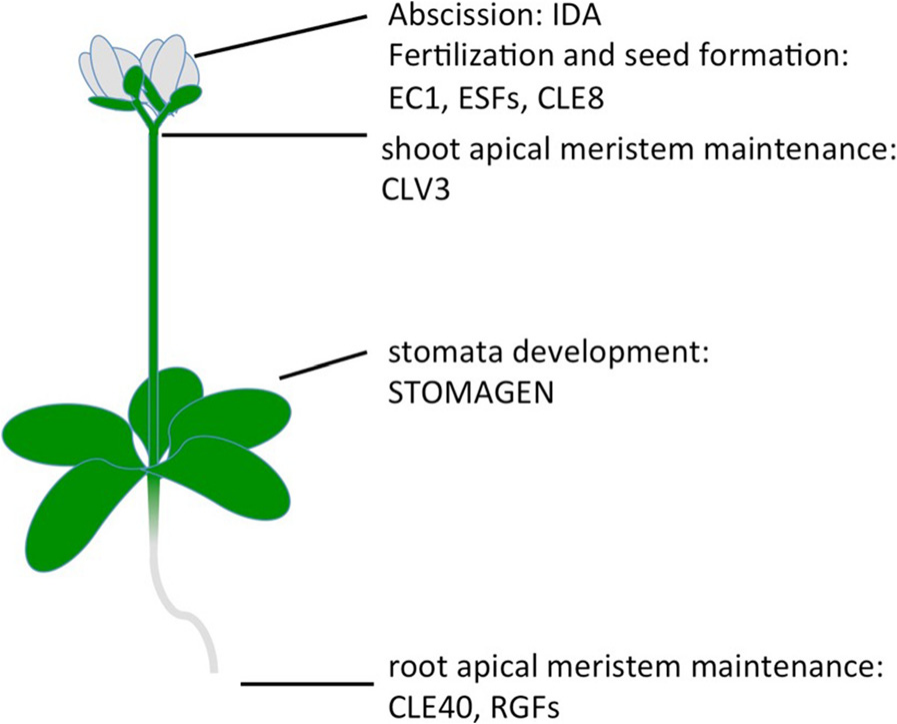
Representatives of signaling peptides involved in developmental processes

Binding of the signaling peptide to the corresponding receptor leads to the activation of various pathways. For example, peptides important in developmental processes regulate the stem cell niches in shoot or root apical meristems (CLV3 and CLE40) or the promotion of abscission (IDA) [8, 10, 42]. The cysteine-rich peptide STOMAGEN is involved in stomata development and overexpression or addition of STOMAGEN increases the number of stomata in plants [27]. Some examples of developmentally important signaling peptides can be found in Fig. 2. Besides functions in developmental processes, signaling peptides are also important in stress responses and symbiotic interactions with microbes [43–45]. As a prime example, the phytosulfonkines (PSKs) may serve to integrate signals from interactions with pathogens and symbionts with the plant’s growth requirements [46].

## Are there any well-studied examples of plant peptides

Yes—for example, several peptide-triggered pathways regulate the maintenance of long-lasting populations of stem cells in meristems, which is a prerequisite for continuous plant development. The feedback loop controlling shoot apical meristem size is a well-studied example of the role of signaling peptides in development. This feedback loop comprises the signaling peptide CLV3, the LRR-RLK CLV1, a receptor heteromer consisting of the RLP CLV2 and the pseudokinase CRN (CORYNE), and the RLK RPK2 (RECEPTOR-LIKE PROTEIN KINASE 2)/TOAD2 (TOADSTOOL 2) [10, 47–50]. Binding of CLV3 to the receptors negatively regulates the expression of *WUS (WUSCHEL)*, which encodes a homeodomain transcription factor. *WUS* is expressed in the organizing center and promotes stem cell identity of cells at the apex of the shoot meristem [51]. WUS thereby positively regulates the expression of stem cell-expressed *CLV3* and thus establishes a negative feedback loop that maintains a relatively stable number of stem cells [51, 52].

The closely related peptide CLE40 controls stem cells in the root meristem [53]. In the distal root meristem, CLE40 acts through the RLK ARABIDOPSIS CRINKLY4 (ACR4), which is structurally unrelated to LRR-RLKs, and also the LRR-RLK CLV1 to promote cell differentiation [42]. In the proximal meristem, CLE40 signaling requires the LRR-RLP CLV2 in a complex with CRN to inhibit or delay cell differentiation. In both parts of the root meristem, CLE40 signaling controls the expression of several phytohormone biosynthetic genes and of stem cell-specific transcription factors [54].

Another example of well-characterized peptide signaling pathways is the IDA peptide-triggered pathway. IDA regulates the separation of cells by inducing degradation of the cell wall during floral organ abscission [8]. The predicted IDA family peptides share a conserved 12 amino acid PIP motif close to their C-termini. The mature IDA peptide consists only of this 12 amino acid motif, contains hydroxyprolinated residues, and shows high activity in a bioassay based on measuring the release of reactive oxygen species [55]. In the abscission zone, IDA interacts with the RLKs HAESA (HAE) and HAESA-LIKE2 (HSL2) [56]. These receptors form complexes with SERKs and trigger the sequential activation of a MAP kinase cascade consisting of MKK4, MKK5, MPK3, and MPK6 [57], and the release of reactive oxygen species via RESPIRATORY BURST OXIDASE HOMOLOGS (RBOH) activity [55, 58, 59]. IDA signaling leads to the suppression of the transcription factor KNAT1 (KNOTTED-LIKE FROM ARABIDOPSIS THALIANA1), which restricts the related TFs KNAT2 and KNAT6 and thereby controls the expression of genes involved in cell separation [60].

**Fig. 3:**
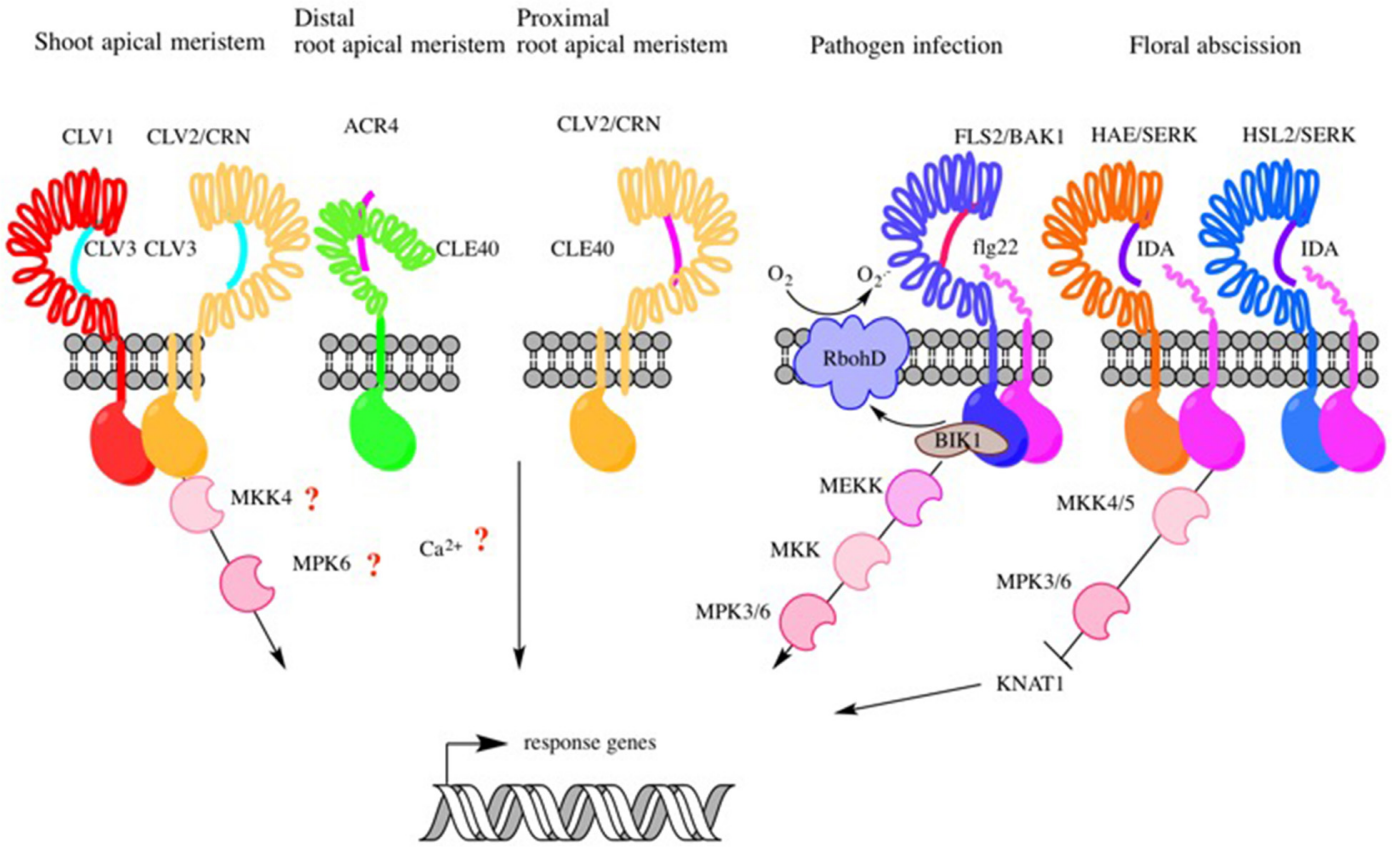
Overview of peptide perception by different receptors. CLV3 can be perceived by CLV1 or CLV2/CRN and RPK2/TOAD2 (not shown in the figure) in the shoot apical meristem. CLE40 is recognized by different receptors in the proximal and distal root meristem (CLV2/CRN, ACR4, and CLV1). The downstream signaling components of these CLE pathways are not yet known. MKK5, MPK6, and calcium signaling might play a role. The pathogen infection signaling pathway mediated by flg22 binding to FLS2 is better understood. Binding of flg22 to the receptor complex FLS2/BAK1 leads to activation of BIK1, which then activates downstream components such as RbohD or MAPKs. Floral abscission is mediated by the signaling peptide IDA, which interacts with the receptors HAE and HSL2. Receptor activation leads to binding to SERKs and to the downstream signaling cascade which involves MKK4/5, MPK3/6, and KNAT1

Signaling peptides are also involved in fertilization and seed formation. EC1 (EGG CELL 1) is a cysteine-rich polypeptide that is important during double fertilization for priming sperm cell activation [61]. Together with four *EC1*-like genes, *EC1* is essential for sperm cell fusion to the female gametes. The cysteine-rich EMBRYO SURROUNDING FACTOR 1 (ESF1) peptides act redundantly to *EC1* [62]. CLE8 is a small signaling peptide that is involved in *Arabidopsis* embryogenesis. *cle8* mutant plants show a high percentage of defective seeds with phenotypes including wrinkled seeds or seeds aborted at early developmental stages [63]. *CLE8* is expressed in the early embryo and induces expression of the WUS related transcription factor WOX8 in suspensor cells, leading to a CLE8–WOX8 regulatory module that organizes suspensor and endosperm development. An overview of the pathways induced by CLV3, CLE40, and IDA is shown in Fig. 3.

## How were the peptide-signaling pathways that control plant development analyzed?

Many of them were first observed by knock-out mutant phenotypes. Later, related family members were often studied by overexpression analysis. Bioinformatic analysis of plant genomes allowed for prediction and identification of further families. Today, it is estimated that around 1800 peptides are encoded by the *Arabidopsis* genome. Since signaling peptides might be able to travel long distances within the plant and interact with multiple receptors, the identification of the corresponding receptor is not always easy. In 2014 Tabata et al. [64] published a novel approach to identify peptide–receptor complexes. They generated an expression library of *Arabidopsis* LRR-RLKs by overexpressing the proteins in tobacco BY-2 cells. Using photoaffinity-labeled peptides, they could identify new receptors that interact with the peptide. This presented technique is promising for identification of so far unknown receptors for several signaling peptides.

## What about pathogen perception?

Some signaling peptides are involved in pathogen perception. One of the best studied pathogen response pathways is the LRR-RLK FLAGELLIN SENSING2 (FLS2)-mediated pathway in the innate immune response. FLS2 plays a critical role in sensing pathogens by binding to bacterial flagellin [65]. Upon flagellin binding, FLS2 interacts with the LRR-RLK BAK1, which leads to intracellular calcium signaling and activation of downstream responses [66, 67]. The receptor-like cytoplasmatic kinase (RLCK) BRI1-ASSOCIATED KINASE1/SOMATIC EMBRYO RECEPTOR KINASE3 (BIK1) interacts with FLS2 and BAK1 and is phosphorylated by BAK1 upon flagellin binding. BIK1 then dissociates from the complex and participates in downstream phosphorylation signaling cascades [68]. BIK1 directly phosphorylates the NADPH oxidase RbohD, which leads to a Ca^2+^ influx-dependent oxidative burst by the production of reactive oxygen species (ROS) [69]. Other immediate downstream components of this signaling pathway are, for example, the Ca^2+^ influx channels ACA8 and ACA10 (AUTOINHIBITED Ca^2+^ ATPase) that interact with FLS2 after flg22 binding and aggregate in lipid rafts [70]. The activation of MAPKs upon flagellin binding to FLS2 is not dependent on BIK1; hence, FLS2 activation leads to the activation of separable downstream pathways including Ca^2+^ transients [71]. An overview of LRR-RLK FLS2 signaling pathway is represented in Fig. 3.

Besides developmental processes, CLE peptides are also involved in nematode attacks. Nematodes secrete various CLE-like effector proteins, which are injected into the host plant during the parasitic cycle. For example, a CLE-like gene was identified in *Heterodera glycines*, a soybean cyst nematode, which is thought to be an effector gene required for pathogenicity [72]. Several nematode CLEs can mimic endogenous *Arabidopsis* CLEs and act as ligands for *Arabidopsis* receptors, thereby ensuring successful infection by the nematode and development of the syncytium, a multinucleate cell formed by fusion of several plant cells, on which it feeds [73].

## Is there crosstalk between plant peptide signaling pathways?

There is potential for crosstalk because highly similar CLE genes are clustered (CLE4/5/6/7) and some of them even encode peptides with the same amino acid sequence. Due to this probably very recent gene duplication, they might still act redundantly [19]. Indeed, addition of various CLE peptides often leads to similar phenotypes, indicating redundancy but also possible crosstalk between signaling pathways. For example, CLE42 and CLE41/CLE44 inhibit tracheary element differentiation but do not inhibit root growth [35]. In contrast, overexpression of many other *CLE* genes suppresses root growth.

There is also the potential for crosstalk at the receptor level: CLV3 signaling leads to the repression of not only *WUS* but also the BARELY ANY MERISTEM (*BAM*) gene, which encodes a CLV1-related RLK. In *clv1* mutant backgrounds, BAM1 is upregulated and can perceive the CLV3 ligand. The normal ligand for BAM1 has not yet been identified but is likely also a CLE peptide. Nimchuk et al. [74] suggested that *BAM* expression in the shoot apical meristem could contribute to the robustness of the CLV network against perturbations.

A much-discussed publication in 2011 reported that CLV3 can trigger the innate immune response via binding to the FLS2 receptor [75], suggesting crosstalk between developmental and pathogen response pathways. The authors indicated that such a mechanism may prime cells at their point of origin, the stem cell zone, for future encounters with pathogenic bacteria. However, the CLE40 peptide, which is closely related to CLV3, could not initiate any response mediated by FLS2. Sengonzac et al. [76] then meticulously tested the effect of CLV3 on FLS2 in *Arabidopsis* mesophyll protoplasts and seedlings but could not detect any immune response, leading them and others to the conclusion that FLS2 is not able to perceive CLV3 as a signal.

## …and how is specificity of plant peptide signaling generated?

Specific functions and interactions of signaling peptides are generated by their regional expression pattern in the plant and their distinct binding properties to their corresponding receptors. Furthermore, the localized expression of the receptors and availability for only some signaling peptides restrict their signaling activity.

## How far do peptides travel in the plant?

Long-distance signaling has been shown for several signaling peptides in plants. In legumes, homologues of CLV3 travel from the root to the shoot to regulate nodule number [77]. The energy-consuming formation of nodules has to be strictly regulated. The shoot receives a signal from the root, which is generated upon root nodulation. In *Lotus japanicus*, HYPERNODULATION ABERRANT ROOT FORMATION1 (HAR1), a CLV1 like receptor kinase, is required in the shoot and binds the CLE-root signal 2 peptide (RS2), which is generated in the root [77, 78]. CLE-RS2 was shown to be transported via the xylem, but the mechanism for how the peptide is loaded into the xylem remains unclear [79]. Transport of CLE2, 3, 4, and 7 via the apoplast is required for expansion of the root system in nitrogen-poor environments [80].

## Are plant peptides relevant for future agriculture?

Analyzing the effect of peptides on plant development can be beneficial for future agriculture. Identification of novel signaling peptides that influence meristem size may help finding mutants which lead to an increased yield. The search for mutant plants that are carrying mutations in the genes for the signaling peptides or their corresponding receptors can be improved by ongoing research in this area. Mutations in the maize orthologue of CLV1 *thick tassel dwarf1* (*td1*) are known to affect female and male inflorescence ear, which ultimately gives rise to seeds [81]. The female inflorescence shows more kernels and the male an increased spikelet density. The maize LRR-RLP FASCIATED EAR3 (FEA3) functions in stem cell control and is repressed by WUS. Je et al. [82] proposed a feedback model where a CLE peptide signal moves from the organ primordia to the shoot apical meristem and is then perceived by FEA3 to regulate meristem size. Additionally, they could also show that weak alleles of *fea3* lead to a significant increase in yield in field experiments.

Overall, we have gained information on only a tiny number of peptide-triggered signaling pathways in plants; many hundreds still await analysis, and these peptides may provide us with very precise tools to modify plant architecture and development for crop improvement.
